# Limits to in vivo fate changes of epithelia in thymus and parathyroid by ectopic expression of transcription factors Gcm2 and Foxn1

**DOI:** 10.1038/s41598-022-17844-2

**Published:** 2022-08-08

**Authors:** Daisuke Nagakubo, Mayumi Hirakawa, Norimasa Iwanami, Thomas Boehm

**Affiliations:** 1 Department of Developmental Immunology, Max Planck Institute of Immunobiology and Epigenetics, Stuebeweg 51, 79108 Freiburg, Germany; 2 Present Address: Division of Health and Hygienic Sciences, Faculty of Pharmaceutical Sciences, Himeji Dokkyo University, 7-2-1 Kamiohno, Himeji, Hyogo 670-8524 Japan; 3 Present Address: Division of Immunology and Allergy, Research Institute for Biomedical Sciences, Tokyo University of Science, 2669 Yamazaki, Noda-City, Chiba 278-0022 Japan; 4 Present Address: Center for Bioscience Research and Education, Utsunomiya University, Utsunomiya, Tochigi 321–8505 Japan

**Keywords:** Developmental biology, Immunology

## Abstract

The development of the parathyroid and the thymus from the third pharyngeal pouch depends on the activities of the Gcm2 and Foxn1 transcription factors, respectively, whose expression domains sharply demarcate two regions in the developing third pharyngeal pouch. Here, we have generated novel mouse models to examine whether ectopic co-expression of *Gcm2* in the thymic epithelium and of *Foxn1* in the parathyroid perturbs the establishment of organ fates in vivo. Expression of *Gcm2* in the thymic rudiment does not activate a parathyroid-specific expression programme, even in the absence of Foxn1 activity. Co-expression of *Foxn1* in the parathyroid fails to impose thymopoietic capacity. We conclude that the actions of Foxn1 and Gcm2 transcription factors are cell context-dependent and that they each require permissive transcription factor landscapes in order to successfully interfere with organ-specific cell fate.

## Introduction

**Figure 1:**
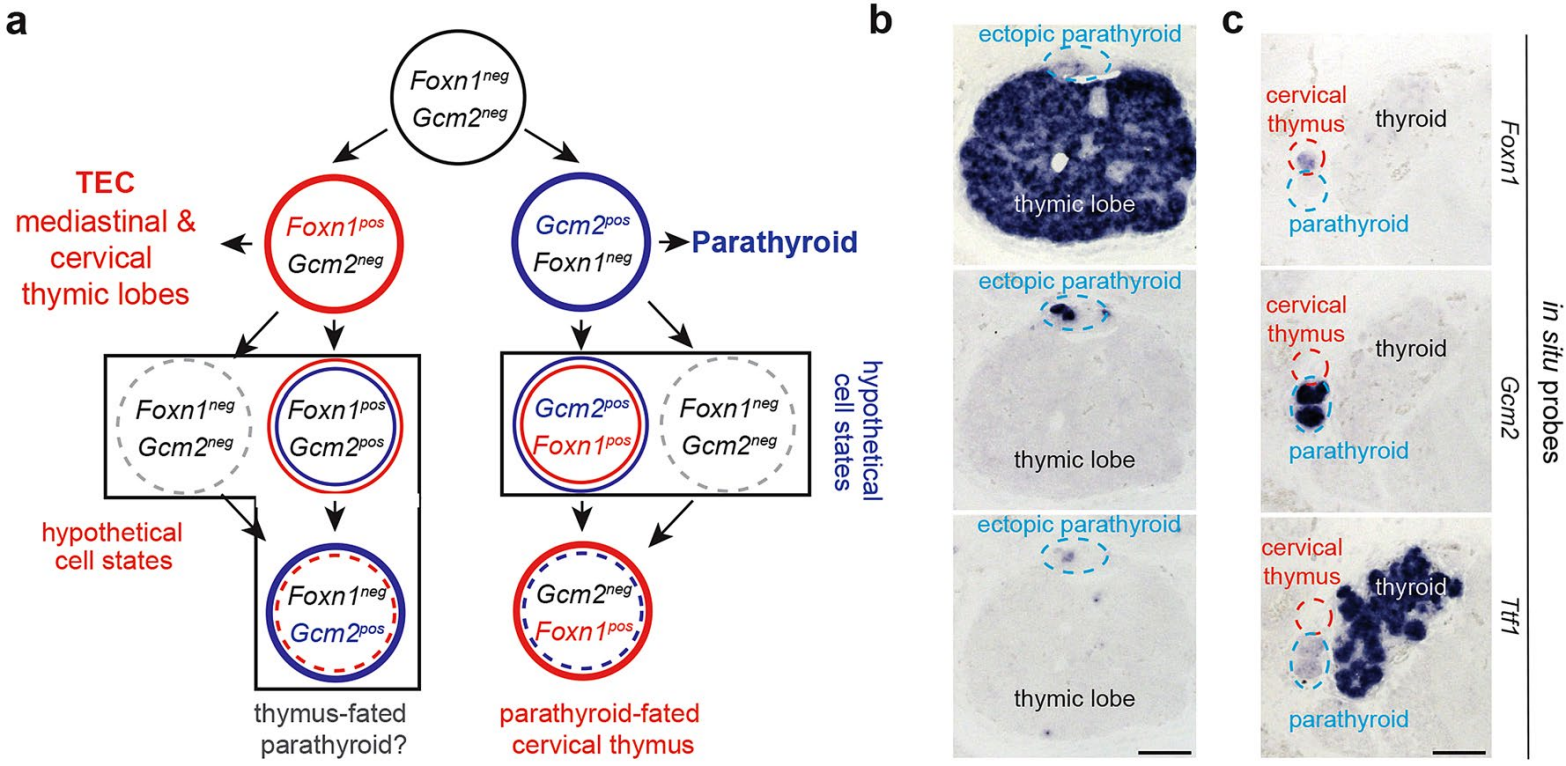
Developmental trajectories of thymus and parathyroid. (**a**) In mice, the third pharyngeal pouch initially consists of undifferentiated epithelium that expresses neither *Foxn1* nor *Gcm2*. At a later stage, two distinct domains emerge, one expressing *Foxn1*, the other *Gcm2*. Whereas the former goes on to form the epithelial component (TEC) of mediastinal and cervical thymic lobes, the latter develops into the parathyroid. Upon further (hypothetical) changes in cell states, some parathyroid-fated cells may transdifferentiate into a second kind of cervical thymus, referred to as parathyroid-fated cervical thymus (see text for details). Evidence for the existence of analogous thymus-fated parathyroid tissue has not yet been found. (**b**) RNA in situ hybridisation patterns identify and distinguish thymus and parathyroid tissues. In some instances, ectopic parathyroid tissue remains attached to the thymic lobe as a result of incomplete separation of parathyroid and thymus anlagen during embryogenesis, as described earlier^20^; in the present context, these ectopic patches of cells serve as a positive technical control for the lack of expression of *Pth* in the thymic epithelium, but do not represent fate-converted tissue (c.f., Fig. 2 in^20^). (**c**) RNA in situ hybridisation patterns identify and distinguish thyroid and parathyroid tissues. For (**b**), and (**c**), serial sections of E14.5 embryos were hybridized with the indicated gene-specific probes. Data representative of ten embryos.

In mice, several organs develop from the epithelial lining of the pharynx, such as the thyroid, the ultimobranchial bodies, the parathyroid, and the thymus. The anlagen of the parathyroid and the thymus emerge in the third pharyngeal pouch, the parathyroid from the dorso-cranial aspects of the pouch, the thymus from the caudo-ventral parts. The proper development of the future organs depends on the activities of transcription factors Gcm2 for the parathyroid^1–3^ and Foxn1 for the thymus^4,5^ (Fig. 1a). The expression domains of the two genes mark complementary regions in the developing third pouch even before morphological differences in the tissue become recognizable^6^. However, the phenotypes of mice lacking *Gcm2*^1–3^ or *Foxn1*^4,5,7^ suggest that these transcription factors are not required for the formation of organ primordia. Instead, they have been shown to play key roles at subsequent stages of development and to participate in the execution of organ-specific functional programmes^3,7^. In mice lacking functional *Gcm2*, the parathyroid rudiment forms in the third pouch, but then undergoes apoptotic regression, causing the parathyroid to disappear^1,2,8^. Hence, although *Gcm2* is not required for the initiation of parathyroid development, it is required for the maintenance of this gland; as a result, *Gcm2*-null mice suffer from impaired calcium homeostasis^1,8^. In mammals lacking a functional *Foxn1* gene, the epithelial component of the thymic rudiment forms during embryogenesis; however, in contrast to the situation of *Gcm2*-deficiency in the parathyroid, the *Foxn1*-deficient thymus-fated epithelium persists into adulthood. Although it fails to differentiate properly and hence remains in an immature non-functional state^4,5,7^ and is incapable of attracting haematopoietic progenitor cells, the subsequent re-introduction of *Foxn1* enables the activation of the stalled differentiation programme^9^. *Foxn1* deficiency in mammals thus effectively blocks intrathymic T cell development^10,11^, leading to profound immunodeficiency^4,12,13^. Remarkably, overexpression of *Foxn1* in certain heterologous cell types, such as fibroblasts, has been reported to generate functional thymic epithelial cells^14,15^. This suggests that in permissive cellular environments, Foxn1 can impose a thymic epithelial cell fate.

During embryonic development, the thymic anlage (Fig. 1b) migrates to the upper anterior mediastinum, whereas the parathyroid gland becomes closely associated with the thyroid, which is marked by high levels of expression of the *Ttf1* transcription factor gene (Fig. 1c)^16^. Interestingly, however, ectopic (cervical) thymic tissue can be found in many different locations in the neck region, sometimes even in close proximity to the parathyroid^17,18^ (Fig. 1c). Cervical thymi may arise either from delayed differentiation of an undifferentiated endodermal progenitor pool^18,19^, or from transdifferentiation of parathyroid-fated cells, after downregulation of their parathyroid differentiation programme and subsequent upregulation of *Foxn1* expression^19^ (Fig. 1a). These transfated cervical thymi attract haematopoietic progenitors and induce the initiation of their T cell differentiation programme^19^. It is unclear whether transdifferentiation proceeds through intermediate *Gcm2/Foxn1* double-positive and/or double-negative stages (indicated as hypothetical cell states in Fig. 1a). Considering the final outcome of these differentiation trajectories, we hypothesize that enforced *Foxn1* expression in the parathyroid domain should induce transdifferentiation in post-*Gcm2*-expressing cells (Fig. 1a).

Ectopic parathyroid tissue is also often found in scattered locations in the neck, and indeed in the mediastinum adjacent to or inside thymic lobes (Fig. 1b)^20^; in humans, this presents a significant challenge when extirpation of parathyroid adenomas is required^21^. Ectopic parathyroid tissue may not always result from aberrant migration to the final anatomical location next to the thyroid, but could, in analogy to the situation of the parathyroid-fated cervical thymi, originate from trans-fated thymic epithelium (Fig. 1a). To the best of our knowledge, direct evidence for this kind of a transfating event has not yet been reported.

To begin to experimentally address this developmental conundrum, we have generated mice in which epithelia of the two organ anlagen co-express the two transcription factors. In our scheme, the epithelium in the thymic rudiment additionally expresses *Gcm2* under the control of the *Foxn1* regulatory elements, whereas the parathyroid anlage additionally expresses *Foxn1* under the control of the regulatory elements of the *Gcm2* gene.

## Results

### Strategy for the ectopic expression of *Gcm2* in the expression domain of *Foxn1*

**Figure 2:**
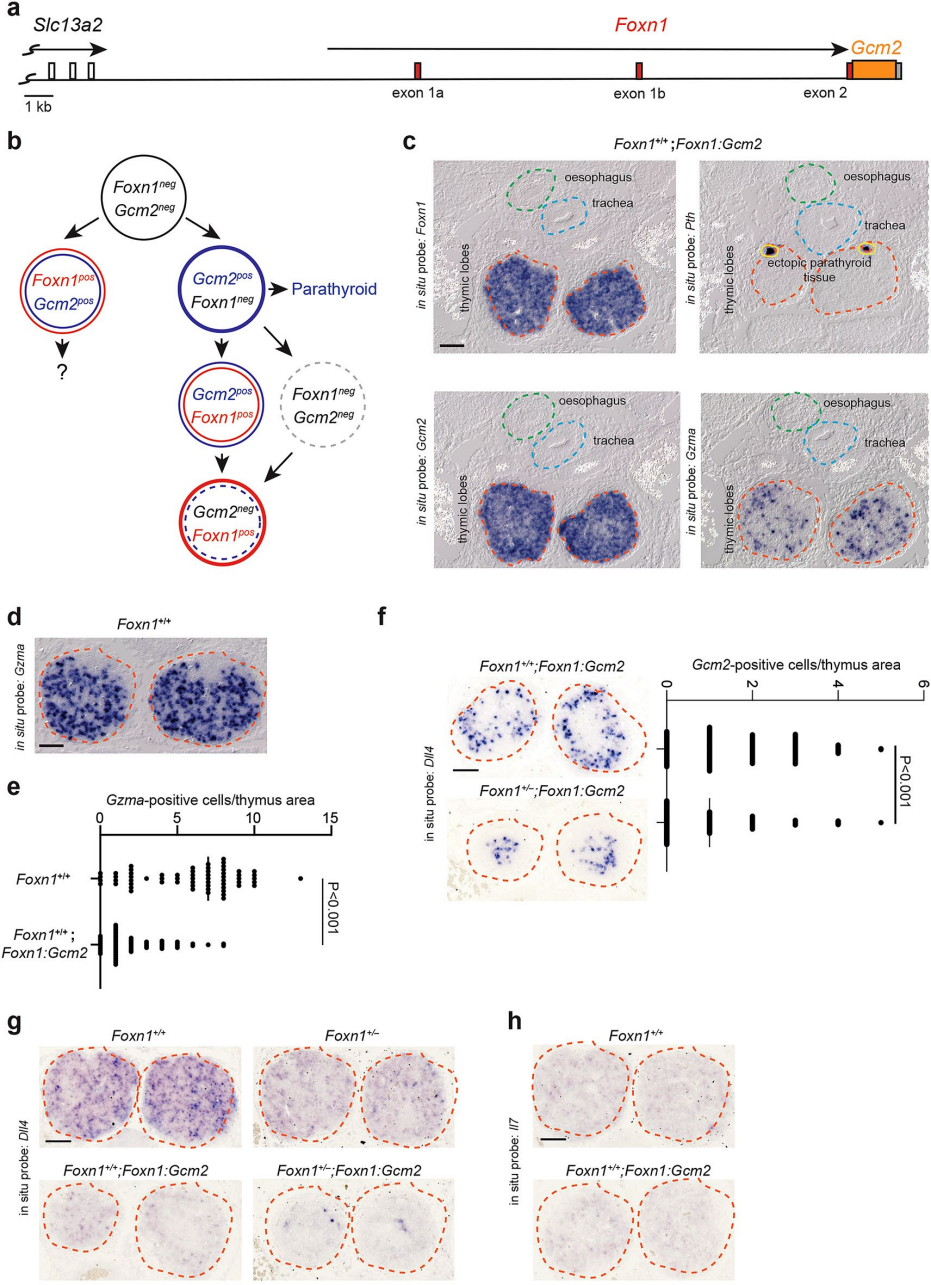
Ectopic expression of *Gcm2* in *Foxn1*-positive epithelial cells of *Foxn1*^+/+^ mice. (**a**) Schematic of the *Foxn1:Gcm2* expression construct. The individual sequence elements are indicated; note that the exonic sequences following the two alternative transcriptional start sites of the *Foxn1* gene (identified as exons 1a and 1b, respectively) both associate with the same splice acceptor site of exon 2. The *Gcm2* cDNA is inserted in exon 2 of the *Foxn1* gene. The presence of the polyadenylation sequence of the *Slc13a2* gene serves as an insulator sequence suppressing insertion site-associated variegated activity of the transgene. (**b**), Developmental trajectories under conditions of ectopic *Gcm2* expression in thymic epithelial cells; note that the development of parathyroid and parathyroid-fated cervical thymi is expected to occur normally. Data representative of two mice. (**c**) RNA in situ hybridisation of E14.5 tissue sections of the upper mediastinum of *Foxn1:Gcm2* transgenic mice with *Foxn1*-, *Gcm2*-, *Pth*-, and *Gzma*-specific probes. Data representative of three mice. (**d**) RNA in situ hybridisation identifies *Gzma*-positive thymocytes in the wild-type thymus. (**e**), Number of *Gzma*-positive cells per thymus area (2,500 μm^2^) of wild-type and *Foxn1:Gcm2* transgenic mice; the median value is 7 cells for the wild-type (5.7903 ± 0.4063; mean ± s.e.m.), and 1 cell for the transgenic tissue (2.1754 ± 0.2789; mean ± s.e.m.) (Welch t-test, two-sided). Two thymic lobes each were analysed. (**f**) RNA in situ hybridisation of E14.5 tissue sections of thymic lobes of *Foxn*1^+/+^ wildtype; Foxn1^+/–^ heterozygous, and *Foxn1*^+/+^;*Foxn1:Gcm2* and *Foxn1*^+/–^;*Foxn1:Gcm2* transgenic mice with a *Dll4*-specific probe. (**g**) RNA in situ hybridisation of E14.5 tissue sections of thymic lobes of *Foxn*1^+/+^;*Foxn1:Gcm2* and *Foxn1*^+/−^;*Foxn1:Gcm2* transgenic mice with a *Gzma*-specific probe (left panel); number of *Gzma*-positive cells per thymus area (2,500 μm^2^) of *Foxn*1^+/+^;*Foxn1:Gcm2* and *Foxn1*^+/−^;*Foxn1:Gcm2* transgenic mice; the median value is 1 cell for *Foxn*1^+/+^;*Foxn1:Gcm2* (1.6061 ± 0.1005; mean ± s.e.m.; 6 thymic lobes analysed), and 1 cell for the *Foxn*1^+/−^;*Foxn1:Gcm2* tissue (0.9589 ± 0.0991; mean ± s.e.m.; 8 thymic lobes analysed) (Welch t-test, two-sided). (**h**), RNA in situ hybridisation of E14.5 tissue sections of thymic lobes of *Foxn*1^+/+^ wildtype and *Foxn1*^+/+^;*Foxn1:Gcm2* transgenic mice with an *Il7*-specific probe. Note that, at this stage of development, *Il7* expression in the wildtype thymus is just above the detection limit^28^; the results are shown here to indicate that *Il7* expression is not elevated in the transgenic situation (see text). Note that results in panels (**e**) and (**g**) come from independent experiments; at these early embryonic stages, T cell development is very dynamic; therefore, slight differences in developmental time may influence the results necessitating the analysis of animals from the same litter as the best possible age-match. No positive signal could be detected with a B-cell specific probe (*Cd19*; not shown). The organ primordia are outlined by dashed lines. Scale bars, 0.1 mm.

In the first set of experiments, we focused on the ectopic expression of *Gcm2* in the *Foxn1* expression domain, which marks the future thymic epithelial microenvironment. Two genetic constellations were explored. In order to avoid complications arising from the known *Foxn1* haploinsufficiency effect^22,23^, we used a transgenic strategy to additionally express *Gcm2* in the *Foxn1* domain. To this end, we placed the *Gcm2* cDNA under the control of the complex regulatory elements of the *Foxn1* gene, encompassed in a ~ 28 kb genomic fragment situated upstream of the *Foxn1* gene (Fig. 2a)^24^. This strategy has been successfully used to express numerous cDNAs in the thymic rudiment (e.g.,^10,17,18,25–29^). In our experiments, the numbers of *Gzma*-expressing thymocytes within the thymus were used as a measure of thymopoietic activity. *Gcm2*-mediated fate changes were assessed by the expression of the parathyroid hormone (*Pth*) gene, one of Gcm2´s key down-stream targets and a characteristic sign of parathyroid fate^30–32^. Thus, RNA in situ hybridisation assays are a sensitive means to determine residual *Foxn1*-driven thymi epithelial function (*Gzma*) and the initiation of the parathyroid-specific expression programme (*Pth*).

### Expression of *Gcm2* in *Foxn1*-sufficient thymic epithelia

In order to assess the effects of *Gcm2* expression in thymic epithelia, we first generated a condition of co-expression of *Foxn1* and *Gcm2* transcription factor genes (Fig. 2b), corresponding to one of the hypothetical cell states illustrated in Fig. 1a. In the *Foxn1*-sufficient background, *Gcm2* expression can be readily detected in the transgenic thymic rudiment (Fig. 2c). The ectopic activity of the *Gcm2* transcription factor gene does not perturb the expression of the *Foxn1* gene (Fig. 2c). Accordingly, at a qualitative level, the *Foxn1*-controlled genetic network remains functional, since thymic colonisation and T cell differentiation occur in the transgenic thymi, as indicated by the presence of cells positive for the T cell-specific hybridisation probe *Gzma* (Fig. 2c). However, compared to wild-type thymic epithelium (Fig. 2d), the thymopoietic activity of the thymic epithelium co-expressing *Gcm2* is reduced several-fold, as indicated by the presence of significantly fewer *Gzma*-positive cells (Fig. 2c–e). This phenotype is accompanied by significantly reduced expression levels of the *Dll4* gene, the key target of Foxn1 required to initiate T cell development via Notch signalling^10^ (Fig. 2f). Of note, the numbers of *Gzma*-positive cells are further reduced when ectopic expression of *Gcm2* occurs in *Foxn1* heterozygous mice (Fig. 2g), indicating that the detrimental effect of *Gcm2* expression on thymopoiesis is magnified under conditions of reduced *Foxn1* activity. However, despite reduced *Dll4* expression levels, *Cd19*-expressing cells indicative of B cell development are not found the transgenic thymic rudiment (not shown), presumably because no concomitant up-regulation of the *Il7* gene is observed (Fig. 2h) that would have resulted in a perturbed ratio of *Il7/Dll4* expression levels that we have previously found to be an important factor for thymic B cell poiesis^27,28^. Importantly, *Gcm2* expression fails to activate its cognate differentiation programme in *Foxn1*-expressing thymic epithelial cells, as seen from the lack of parathormone (*Pth*) gene expression (Fig. 2c). Collectively, these results suggest that although *Gcm2* expression impairs *Foxn1*-mediated thymopoietic activity, this effect is not accompanied by the concurrent activation of a parathyroid-specific differentiation programme.

### Ectopic *Gcm2* expression in *Foxn1*-deficient epithelia

**Figure 3:**
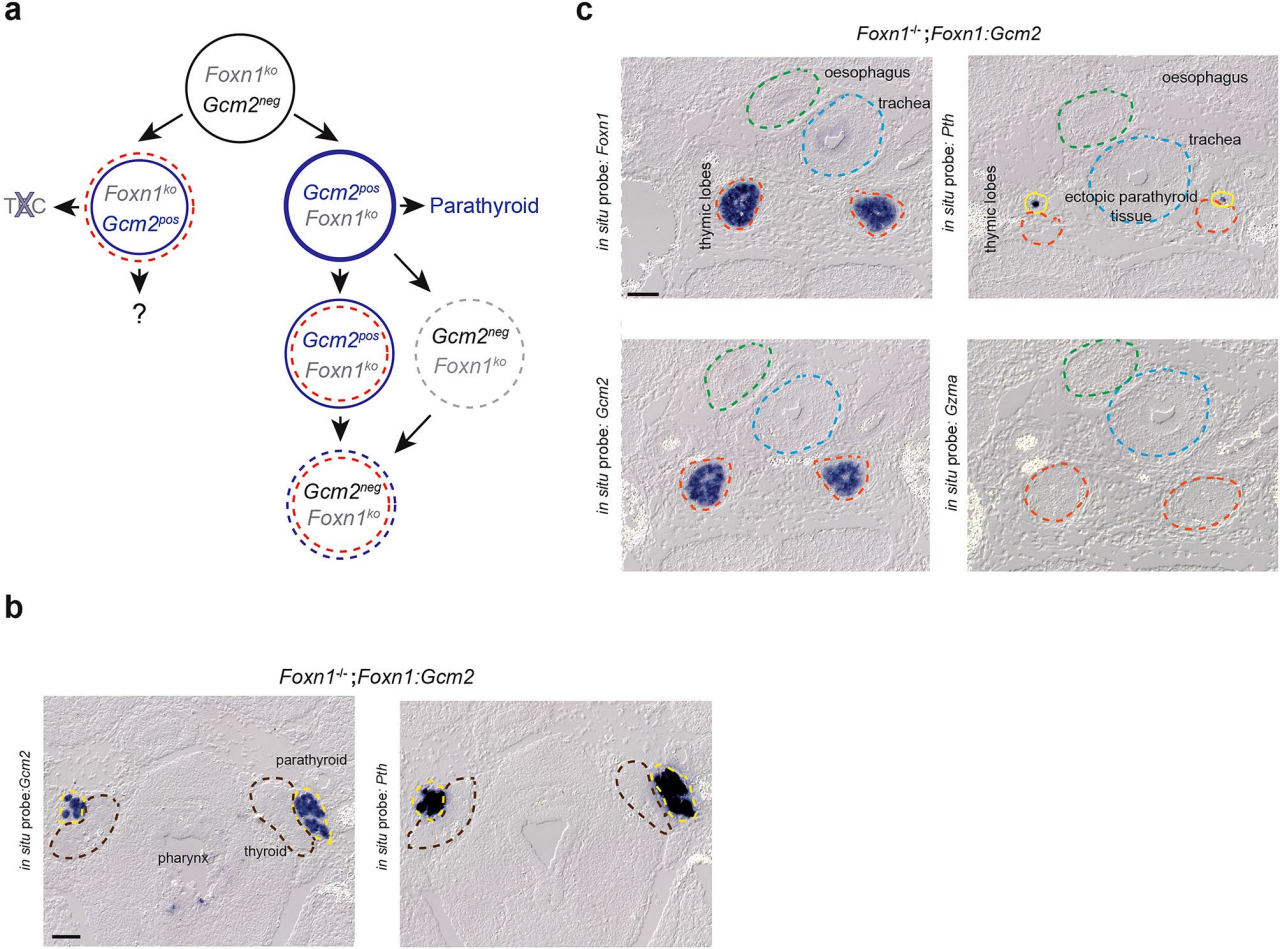
Ectopic expression of *Gcm2* in the thymic epithelium of *Foxn1*-deficient mice. (**a**) Developmental trajectories under conditions of ectopic *Gcm2* expression in *Foxn1*-negative thymic epithelial cells; note that the development of parathyroid is expected to occur normally, whereas that of parathyroid-fated cervical thymi is aborted. (**b**) Control hybridisations of sections of the upper pharynx of the mouse shown in (**c**) using *Gcm2*- and *Pth*-specific probes. Data representative of five embryos. (**c**) RNA in situ hybridisation analysis of the thymic rudiment; note that the *Foxn1*-specific hybridisation probes detects sequences upstream of the insertion site of the *lacZ* gene in the *Foxn1* knock-in allele, such that *Foxn1*-deficient epithelial cells can still be recognized by RNA in situ hybridisation. Tissue sections representative of three embryos. Scale bars, 0.1 mm.

Next, we considered the possibility that *Gcm2* expression may not be able to overrule the function of *Foxn1* in a state of co-activity of these two transcription factors. Therefore, we explored the outcome of ectopically expressing *Gcm2* in *Foxn1*-deficient mice (Fig. 3a). Whereas the development of the parathyroid proceeds normally in this constellation (Fig. 3b), parathyroid-fated cervical thymi are not detectable. Since lack of *Foxn1* activity arrests the thymic epithelium in an undifferentiated state^4,5,7^, *Gcm2*  could exert its function in the absence of an established *Foxn1*-mediated transcriptional programme (Fig. 3a). As expected, *Gcm2* expression is readily detectable in the *Foxn1*-deficient epithelial rudiment without concomitant loss of *Foxn1*  promotor activity (Fig. 3c). Importantly, however, the *Pth* gene again remains transcriptionally silent in the *Gcm2*-expressing thymus-fated epithelia. This result suggests that *Gcm2* expression does not impose the phenotype of the parathyroid onto thymic epithelia, even in the absence of the cognate transcription factor Foxn1.

### Ectopic expression of *Foxn1* in the expression domain of *Gcm2*

**Figure 4:**
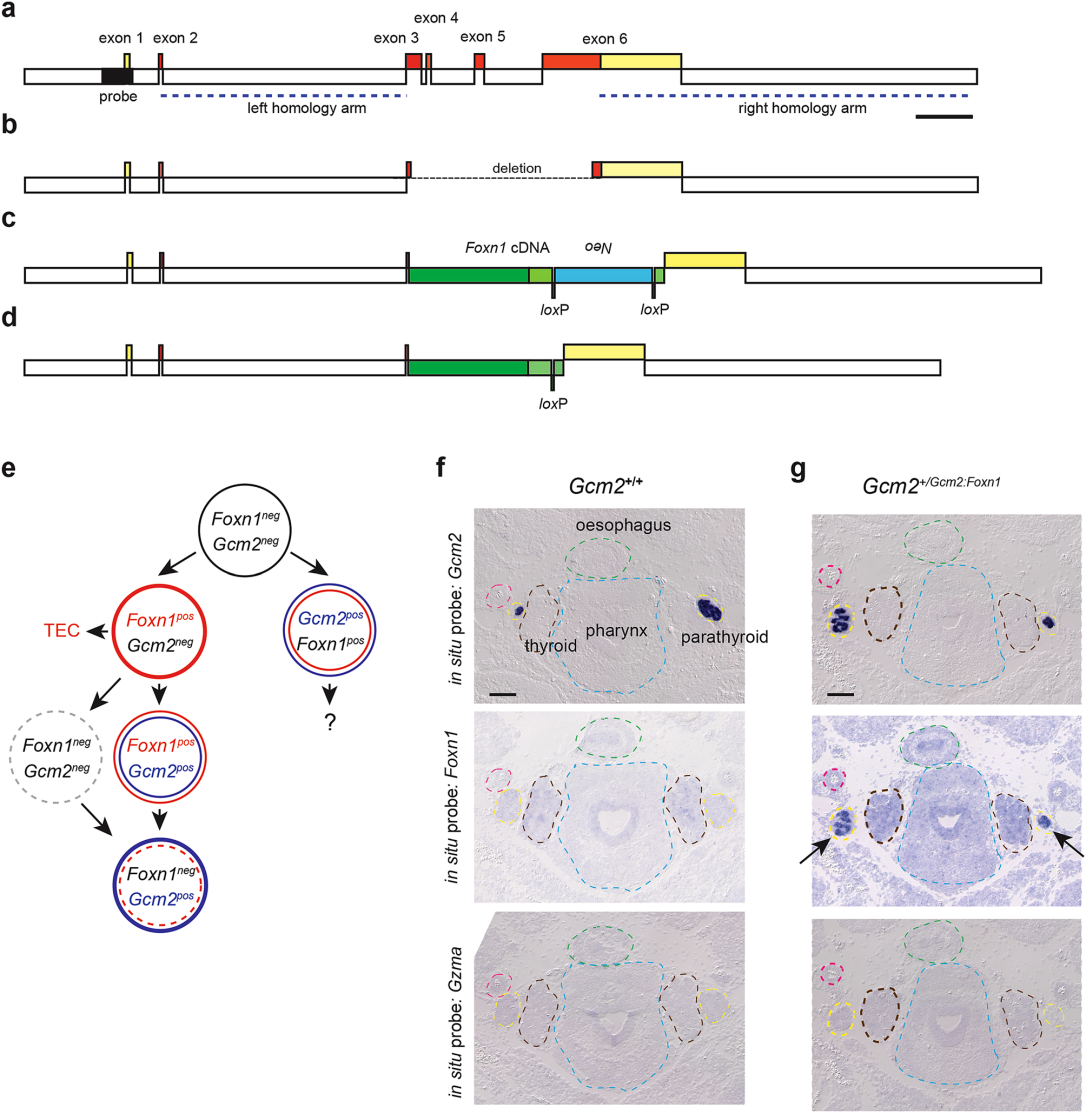
Ectopic expression of *Foxn1* in the parathyroid. (**a–d**) Structure of *Gcm2* alleles generated in this study. (**a**) Structure of the wild-type *Gcm2* locus on mouse chromosome 13. Exons are depicted as red boxes, the 5´- and 3´-untranslated regions are shown in yellow. The initiation codon occurs in exon 2, the stop codon is situated in exon 6. The sequence contained in the fragment used for Southern blot hybridisation is indicated in black and lies outside of the homology arms that were contained in the replacement fragment. The sequences represented in the construct used for homologous recombination are indicated as blue dashed lines. (**b**) Structure of the *Gcm2* null allele. The deleted region extends from exon 3 into exon 6 and is indicated by a dashed line. (**c**) Structure of *Gcm2* locus after homologous recombination. The deleted region is replaced by a cassette consisting of the mouse *Foxn1* cDNA (coding region depicted in dark green; the 3´-untranslated region in light green), and a *Neo* gene (blue), which is positioned in opposite transcriptional orientation; the latter is flanked by two *lox*P sites in tandem orientation (green boxes). (**d**) Structure of *Gcm2* locus after homologous recombination and removal of the *Neo* cassette, which leaves one *lox*P site behind. (**e**) Developmental trajectories under conditions of ectopic *Foxn1* expression in the parathyroid; note that the development of the mediastinal and cervical thymi and the hypothetical thymus-fated parathyroid are expected to occur normally. (**f**) RNA in situ hybridisation of adjacent E14.5 tissue sections of the pharynx of wild-type mice with probes specific for *Gcm2*, *Foxn1*, and *Gzma*. The organ primordia and other anatomical features are outlined by coloured dotted lines. Note that the size of the parathyroid is variable in individual sections. For orientation, the common carotid artery is indicated by red circle on one side of the section. Tissue sections representative of ten mice. (**g**) Hybridisation of sections equivalent to those in (**f**) from mice heterozygous for the *Gcm2:Foxn1* knock-in allele. The *Foxn1*-expressing parathyroid glands are indicated by arrows. Tissue sections representative of 2 mice. Scale bars, 0.1 mm.

In the next set of experiments, we focused on the ectopic expression of *Foxn1* in the *Gcm2* expression domain, which marks the future parathyroid tissue. In order to direct the expression of the Foxn1 transcription factor to the parathyroid, we used CRISPR/Cas9-mediated homologous recombination of a *Foxn1* cDNA sequence into the *Gcm2* locus (Fig. 4a). We aimed at replacing a region of about 3 kb within the *Gcm2* gene, spanning parts of exons 3 and almost the entire exon 6 by a ~ 4.2 kb cassette containing the *Foxn1* cDNA, and a *neo* gene in opposite transcriptional orientation. To this end, we first identified sgRNAs that could be used to specifically and efficiently delete the relevant part of the *Gcm2* gene. In this way, we successfully generated a null allele of *Gcm2* (Fig. 4b). In a second step, we co-injected the selected RNPs, and a targetting construct containing the *Foxn1/neo* cassette into fertilised eggs. In the successfully engineered *Gcm2* allele, the upstream regulatory sequences of the *Gcm2* locus are preserved, as is the large intron (possibly containing regulatory elements) separating exons 2 and 3; the *Foxn1* cDNA was inserted into exon 3 of *Gcm2* in such a way that it preserves the cognate *Foxn1* initiation codon (Fig. 4c). The 3´-untranslated region of the *Foxn1* cDNA harbours a *neo* expression cassette flanked by *lox*P sites, which was removed by breeding mice heterozygous for this allele to a deleter strain expressing *Cre* recombinase in the germ-line^33^, establishing the final *Gcm2:Foxn1* knock-in allele (Fig. 4d). Although the Foxn1-directed transcriptional programme is complex^27,34^,we have previously shown that the expression of only two of the many downstream target genes of Foxn1, those encoding the Notch ligand *Dll4* and the chemokine *Ccxl12* are sufficient to initiate thymopoiesis, as determined by the presence of *Gzma*-positive T cell lineage cells in the thymic rudiment^10^; hence, we considered the presence of *Gzma*-positive cells as a suitable marker of *Foxn1* activity.

### Expression of *Foxn1* in the parathyroid

Using the *Gcm2:Foxn1* knock-in allele, we first generated a condition of co-expression of *Gcm2* and *Foxn1* transcription factor genes in the parathyroid (Fig. 4e). Since *Gcm2* haploinsufficiency has no known detrimental effects on parathyroid function, the parathyroid-fated cells will be simultaneously exposed to both Gcm2-driven and  Foxn1-driven transcriptional programmes (Fig. 4e).

**Figure 5:**
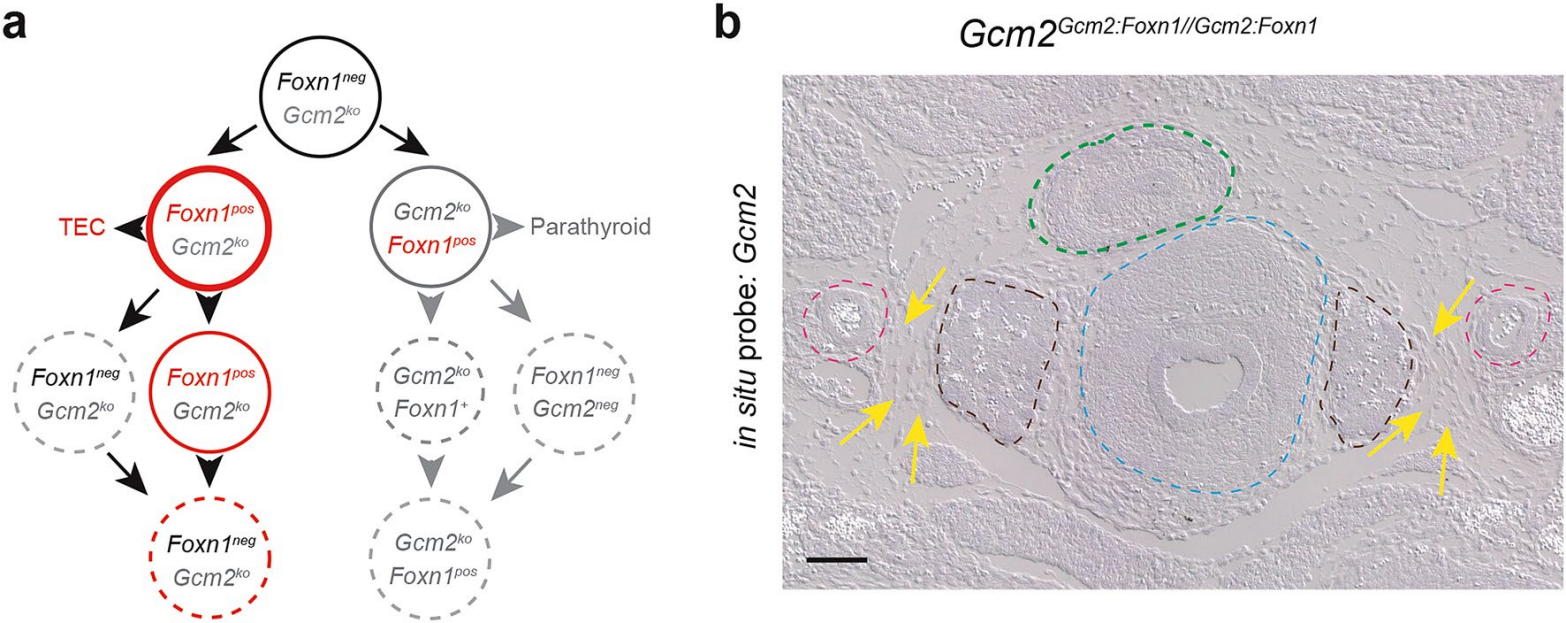
Lack of parathyroid tissue in E14.5 embryos homozygous for the *Gcm2:Foxn1* knock-in allele. (**a**) Developmental trajectories under conditions of ectopic *Foxn1* expression in *Gcm2*-negative cells of the parathyroid; note that at this stage of development, parathyroid primordia are expected to be detectable only if *Foxn1* expression rescues their apoptotic demise that occurs as a result of the lack of Gcm2 activity. (**b**) Failure to detect Gcm2-expressing parathyroid tissue. The sections were hybridised with a probe specific for the 5´-end of *Gcm2,* which is part of the chimaeric transcript emanating from the knock-in allele. The expected location of parathyroid is marked by yellow arrows; the common carotid arteries are indicated by red circles. Tissue sections representative of two mice. Scale bar, 0.1 mm.

To determine whether *Foxn1* expression could be detected in the parathyroid as a sign of successful genome engineering, mouse embryos were analysed at embryonic day 14.5 (E14.5); at this stage, the parathyroid and thymus anlagen have already separated well enough from each other in order to be able to unambiguously identify the distinct anatomical structures in the pharynx. Using an RNA in situ hybridisation assay, we compared wild-type mice (Fig. 4f) and compound heterozygotes carrying one wild-type *Gcm2* allele and the *Gcm2:Foxn1* knock-in allele (Fig. 4g). As expected, *Foxn1* expression in the parathyroid can only be seen in the compound heterozygote—but not in wild-type mice –, whereas *Gcm2* expression is seen in both genotypes (Fig. 4f,g). This result indicates that the *Gcm2:Foxn1* knock-in allele is active as desired and suggests that the activity of the Foxn1 transcription factor does not repress *Gcm2* gene expression. Thus, the results shown in Fig. 2c and Fig. 4g clearly indicate that Gcm2 and Foxn1 transcription factors do not cross-regulate each other, a result that is compatible with distinct signalling pathways upstream of these two genes^35,36^. Importantly, co-expression of cognate (Gcm2) and ectopic (Foxn1) transcription factors in the parathyroid did not induce thymopoietic activity, as shown by the lack, in the parathyroid, of immature thymocytes expressing granzyme A (*Gzma*) (Fig. 4f,g). In a final set of experiments, we explored the outcome of ectopically expressing *Foxn1* in *Gcm2*-deficient mice (Fig. 5a). In this situation, a potential complication arises from the fact that the parathyroid undergoes apoptotic degeneration in *Gcm2* null mice^1,2,8^. Hence, *Foxn1* expression would have to rescue the demise of the developing parathyroid as a pre-requisite to its functional conversion to a thymopoietic tissue. However, we found that *Foxn1* expression did not rescue the degeneration of the parathyroid in the absence of *Gcm2*, as revealed by the absence of *Gcm2*-expressing cells in the vicinity of the thyroid (Fig. 5b). Hence, it appears that the two transcription factors differ in their anti-apoptotic activities in cells of the parathyroid, essentially precluding the analysis of *Foxn1* function in the parathyroid in the absence of *Gcm2* activity.

## Discussion

Our results provide important information about the roles of Foxn1 and Gcm2 transcription factors in the specification of the thymic epithelium and the parathyroid, respectively. Neither co-expression of *Gcm2* and *Foxn1* in the domain of the future parathyroid nor co-expression of *Foxn1* and *Gcm2* in the future thymic rudiment can override the endogenous differentiation programmes. Since both transcription factors are not required for the initial formation of the organ anlagen, this result may have been expected. However, we were encouraged to revisit this issue following the observation that, under special circumstances, Foxn1 can exhibit inductive activity in cell lines, leading to a stable phenotype of thymic epithelial cells even after transplantation into host mice^14,15^. Moreover, lineage tracing experiments have provided evidence that parathyroid-fated epithelia might change their phenotype to become thymopoietic in vivo^19^. Hence, we speculated that, given the functional parallels between Gcm2 and Foxn1 during normal development, overexpression of Gcm2 may have the same inductive activity in a permissive cell type.

The outcomes of our in vivo experiments, though unsuccessful when viewed from the standpoint of fate conversion, nonetheless provide important information on the developmental stability of cellular fates in the two major domains of the third pharyngeal pouch. Collectively, our data support the notions that Gcm2 and Foxn1 function as executors rather than as initiators of cell fate, when their activities are probed in their developmentally most closely related cell types.

In the case of the parathyroid, we were unable to examine a possible inductive capacity of *Foxn1* in *Gcm2*-null mice. Hence, we cannot exclude that Gcm2 is dominant over the activity of Foxn1 and, as a consequence, blocks the conversion of the parathyroid to thymopoietic tissue. With respect to the developmental scenarios (Fig. 1a) underlying the transdifferentiation of parathyroid-fated cells to thymopietic epithelium^19^, we speculate that the survival of transdifferentiated *Gcm2*-negative, *Foxn1*-positive cells is possible because loss of Gcm2 occurs at a stage when its anti-apoptotic function is no longer required. If true, then our results indicate that this sensitive period ends at E15/16, when the transdifferentiated cervical thymi become readily apparent^19^. By contrast, the inductive capacity of *Gcm2* expression could be examined in the thymic epithelium, both in the presence and in the absence of Foxn1 activity, since the thymic rudiment persists in the absence of an intact *Foxn1* gene. However, in both cases, reprogramming of thymic epithelial cells to cells resembling the phenotype of parathyroid tissue failed.

Collectively, the results of the present experiments strongly suggest that both transcription factors require a permissive gene regulatory network in order to establish tissue identity. Indeed, it was shown that expression of *Pth* requires the cooperation of Gcm2, Mafb, and Gata3 transcription factors in cultured cells^30–32^; the latter two genes are not expressed in thymic epithelial cells^37,38^. We anticipate an analogous situation for Foxn1 in the parathyroid environment. Although to the best of our knowledge, no such information is yet available, we presume that Foxn1 also requires certain co-factor(s) to exert inductive capacity. The conclusion of context-dependent activity of Foxn1 is supported by the fact that although *Foxn1* is expressed in thymic epithelial cells, and additionally in suprabasal keratinocytes of the skin, and in the hair follicle^39–41^, it fails to induce thymopoietic activity in the latter two cell types. Only in thymic epithelial cells does it govern the expression of the characteristic thymopoietic programme, such as the Notch ligand Dll4 and the chemokine Cxcl12^10,34^. By contrast, it has been reported that overexpression of *Foxn1* in fibroblasts in vitro converts them to cells with thymopoietic capacity that persists even after transplantation into mice^14,15^. In the light of the present results, we interpret this latter finding to indicate that fibroblasts provide a permissive transcription factor landscape for Foxn1 to gain inductive activity. Of note, fibroblasts originate from a different germ layer than the derivatives of the pharyngeal endoderm, possibly facilitating the fate-changing capacity of the Foxn1 transcription factor. Future work should be directed at defining critical co-factors of Gcm2 and Foxn1, which, depending on cell type, may consist of different combinations of negative and/or positive regulators.

## Methods

### Mice

The following alleles were generated during the course of this work. The *Foxn1:Gcm2* transgene was generated by cloning a 27,970-bp *Foxn1* promoter fragment (Y12488, nucleotides 5680–33,650) upstream of the mouse *Gcm2* cDNA (Genbank accession number NM_008104; nucleotides 35–1549; downstream of the cDNA the bovine growth hormone polyadenylation sequence (5´- GCCATCTGTTGTTTGCCCCTCCCCCGTGCCTTCCTTGACCCTGGAAGGTGCCACTCCCACTGTCCTTTCCTAATAAAATGAGGAAATTGCATCGCATTGTCTGAGTAGGTGTCATTCTATTCTGGGGGGTGGGGTGGGGCAGGACAGCAAGGGGGAGGATTGGGAAGACAATAGCAGGCATGCTGGGGATGCGGTGGGCTCTATGG) was inserted^29^ (Fig. 2a). The construct was linearized and injected into fertilized eggs of FVB mice to generate the FVB/N-tg(Foxn1-Gcm2)1^Tbo^/Mpie transgenic line. A *Gcm2* null allele (FVB/N-*Gcm2*^*em1Tbo*^/Mpie) was generated by CRISPR/Cas9-mediated internal deletion of nucleotides 124,954 to 127,882 in Genbank accession number AC158538.2 in the *Gcm2* gene using two sgRNAs (nucleotides 124,953 to 124,971; nucleotides 127,878 to 127,896 in Genbank accession number AC158538.2) as described^42^; note that nucleotides 125,018 to 125,035 in Genbank accession number AC158538.2 are retained in the deletion. A *Gcm2:Foxn1* knock-in allele (FVB/N-*Gcm2*^*em1(Foxn1)Tbo*^/Mpie) was generated by homologous recombination in fertilized oocytes primed by simultaneous CRISPR/Cas9-induced internal deletion as described for the *Gcm2* null allele. The left homology arm of the construct contained nucleotides 120,953 to 124,925; the right homology arm spanned nucleotides 128,029 to 134,119 in Genbank accession number AC158538.2. Sandwiched between the two homology arms are positioned mouse *Foxn1* cDNA sequences (nucleotides 95 to 2,417 in Genbank accession number XM_017314286.2), followed by a loxP site (nucleotides 7,256–7,320 in Genbank accession number EF591490.1), a poly(A) signal sequence (nucleotides 6,608 to 6,661 in Genbank accession number AJ627603.1), a *Neo* expression cassette (nucleotides 2,400 to 3,915 in Genbank accession number MK802894.1), a loxP site (nucleotides 52,030 to 52,063 in CP049847.1.). The structures of these two new alleles are described in Fig. 4. The germline Cre-deleter line (*B6.C-Tg(CMV-cre)1Cgn/J*^33^ and the *Foxn1*-deficient line (Foxn1^tm1Tbo^)^7^ were described earlier. Timed matings were used to retrieve embryos at embryonic day (E)14.5; the day of the plug was counted as day E0.5 of gestation. Mice were kept in the animal facility of the Max Planck Institute of Immunobiology and Epigenetics under specific pathogen-free conditions.

Ethics declaration. All experimental protocols were approved by the ethics committee of the Max Planck Institute of Immunbiology and Epigenetics, under license permit 35–9185.81/G-14/57 issued by the Regierungspräsidium Freiburg, Germany. All methods and procedures were carried out in accordance with the relevant guidelines and regulations. All methods are reported in accordance with ARRIVE guidelines. The following information is given in the manuscript: genotypes of animals, groups of animals being compared, sample size, outcome measures, statistical methods, and relevant experimental procedures. No animal was excluded from the study, randomization was not performed, and experimenters were not blinded.

### Genotyping

The following primer combinations were used to genotype the mice used in this study. *Gcm2* wild-type allele: 5´-ccatcttttcccaggaaccaa; 5´-agttggtgacagggtatccgc (amplicon size 465 bp). *Gcm2* null allele: 5´- ccatcttttcccaggaaccaa; 5´-ttgtcaaagctaaagggctgc (amplicon size 200 bp, spanning the deletion). Validation of *Gcm2:Foxn1* knock-in allele by long range PCR: first round, 5´-aaggtgcttctgttcagagggatgc; 5´-atggaaggcaggctgagaagaacag (amplicon size 5,346 bp); second round, 5´-acccacagatgcctcaggtaag; 5´-tgcaccaagcctctgctggga (amplicon size 4,587 bp) under conditions optimal for long-range PCR (Roche). Presence of the knock-in cassette and its correct insertion into the *Gcm2* locus can also be ascertained by Southern blot hybridization using a probe spanning nucleotides 119,822 to 120,317 in Genbank accession number AC158538.2. In subsequent matings, the *Gcm2:Foxn1* knock-in allele was traced using: 5´-cccagactcccctgccccac; 5´-ggtgtcctctctggagggccg (amplicon size 84 bp for the inserted cDNA, 481 bp for the endogenous *Foxn1* gene which serves as internal control for the reaction); in addition, the absence of an internal *Gcm2* sequence (replaced by the knock-in cassette) was examined by 5´-ctttgccatcttttcccaggaac; 5´-caccatttctgccctcactttg (amplicon size 291 bp). *Cre*: 5´-tgcatgatctccggtattga; 5´-cgtactgacggtgggagaat (amplicon size 374 bp); *Foxn1:lacZ* knock-in allele: multiplex assay using 5´-tgcaccaagcctctgctggga; 5´-ctgtgaactcagccatactc; 5´-tcgccttcttgacgagttct (wild-type amplicon size 500 bp; knock-in amplicon size 230 bp). The *Foxn1:Gcm2* transgene can be detected using primers 5´-cattgtagctggctttcttcgag and 5´-cttgtcacagatggctggcctcagc (amplicon size 330 bp).

### RNA in situ hybridisation

Gene-specific probes for *Gcm2*, *Pth*, *Gzma*, and *Foxn1* and conditions for RNA in situ hybridisation have been described^25,26^; details of *Dll4* and *Il7* probes can be found in (ref. 10); B cells were detected using a *Cd19* probe corresponding to nucleotides 1155–1886 of Genbank accession number NM_001357091.1. The *Gcm2* probe recognizing the 5´-end of the mRNA (that is, upstream of the insertion site in the knock-in allele) consists of nucleotides 1229 in Genbank accession number XM_017315354.

### Image analysis

Images were acquired on Zeiss microscopes (Axioplan 2 or Imager Z1 with ApoTome attachment) equipped with AxioCam MRc 5 cameras.

### Statistical analysis and reproducibility

Two tailed t-tests were used to determine the significance levels of the differences between the means of two independent samples, considering equal or unequal variances as determined by the F-test.

## Data Availability

All data supporting the conclusions of this paper are included.
